# Supplementary material for: Phytoplankton diversity and chemotaxonomy in contrasting North Pacific ecosystems
Source: PeerJ. 2023 Jan 3;11:e14501. doi: 10.7717/peerj.14501 (PMC9817951; doi:10.7717/peerj.14501)
Supplement: Supplemental Information 1 — (A) SIMPROF global test on Bray–Curtis similarity matrix of phytoplankton abundances. Pi value is 3.685 (vertical dotted line) with significance level of 0.1% (number of permutations is 999. (B) RELATE analysis between phytoplankton abundances and pigment concentrations showing Spearman rank correlation (Rho) of 0.326 (vertical dotted line) with significance level of 0,1% (number of permutations is 999). [file peerj-11-14501-s001.pdf]

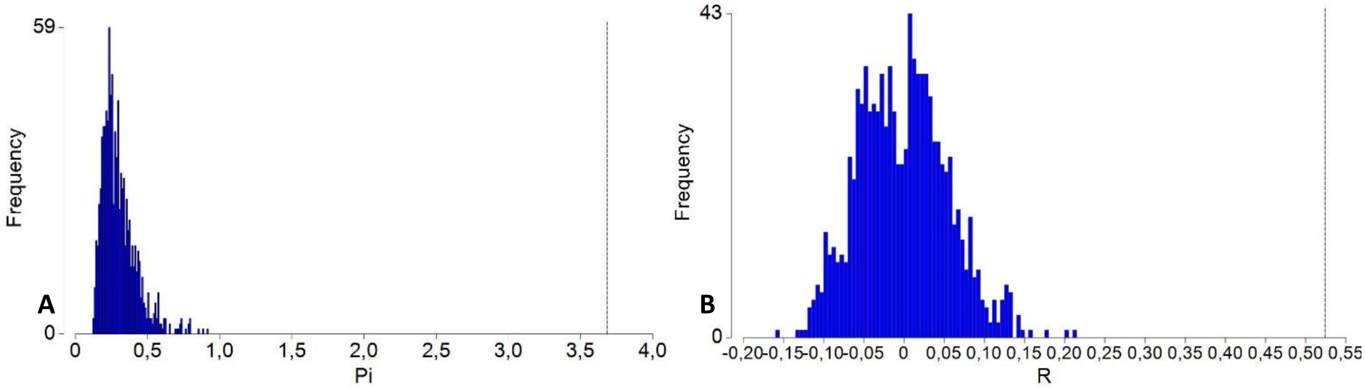

Figure S1. Statistical analysis of phytoplantkon abundance data and pigment concentrations: (**a**) SIMPROF global test on Bray-Curtis similarity matrix of phytoplankton abundances. Pi value is 3.685 (vertical dotted line) with significance level of 0.1%. Number of permutations is 999. Line is out of the plotted area which means that the structure marked with full lines in the dendrogram (Fig 2.) is significant.; (**b**) RELATE analysis between phytoplankton abundances and pigment concentrations showing Spearman rank correlation (Rho) of 0.326 (verical dotted line) with significance level of 0,1 % (number of permutations is 999).
