# Supplementary material for: Phytoplankton diversity and chemotaxonomy in contrasting North Pacific ecosystems
Source: PeerJ. 2023 Jan 3;11:e14501. doi: 10.7717/peerj.14501 (PMC9817951; doi:10.7717/peerj.14501)
Supplement: Supplemental Information 2 — Taxa marked with an asterisk were present only in net samples, SEM abbreviation shows species identified with scanning electron microscope. [file peerj-11-14501-s002.docx]

Table S1. List of taxa/groups determined by the Utermöhl method and recorded in net samples (20 µm). Taxa marked with an asterisk were present only in net samples, SEM abbreviation shows species identified with scanning electron microscope

| Diatoms |
| --- |
| *Alveus marinus* (Grunow) Kaczmarska & Fryxell |
| *Amphora sp.* |
| *Asterolampra marylandica* Ehrenberg * |
| *Asteromphalus heptactis* (Brébisson) Ralfs* |
| *Bacteriastrum comosum* Pavillard |
| *Bacteriastrum furcatum* Shadbolt |
| *Bacteriastrum minus* G.Karsten* |
| *Bacteriastrum biconicum* Pavillard* |
| *Bacteriastrum* spp.* |
| *Caloneis robusta*Cleve |
| *Chaetoceros aequatorialis* Cleve* |
| *Chaetoceros affinis* Lauder |
| *Chaetoceros atlanticus var. neapolitanus* (Schroeder) Hustedt |
| *Chaetoceros coarctatus* Lauder* |
| *Chaetoceros concavicornis* L.A.Mangin* |
| *Chaetoceros constrictus* Gran |
| *Chaetoceros contortus* F.Schütt |
| *Chaetoceros convolutus* Castracane |
| *Chaetoceros curvisetus* Cleve* |
| *Chaetoceros dadayi* Pavillard* |
| *Chaetoceros debilis* Cleve |
| *Chaetoceros decipiens* Cleve |
| *Chaetoceros didymus* Ehrenberg* |
| *Chaetoceros densus* (Cleve) Cleve |
| *Chaetoceros diversus* Cleve* |
| *Chaetoceros indicus* Karsten* |
| *Chaetoceros laciniosus* F.Schütt |
| *Chaetoceros lauderi/teres* Ralfs ex Lauder/Cleve |
| *Chaetoceros messanensis* Castracane |
| *Chaetoceros perpusillus* Cleve |
| *Chaetoceros peruvianus* Brightwell |
| *Chaetoceros pseudoaurivillii* J.Ikari* |
| *Chaetoceros pseudobrevi* Pavillard |
| *Chaetoceros pseudodichaeta* J.Ikari |
| *Chaetoceros pseudosymmetricus* E.Steemann Nielsen* |
| *Chaetoceros radicans* F.Schütt |
| *Chaetoceros simplex* Ostenfeld |
| *Chaetoceros socialis* H.S.Lauder |
| *Chaetoceros* spp.* |
| *Chaetoceros tetrastichon* Cleve |
| *Climacodium biconcavum* Cleve |
| *(Continued on next page)* |
| **Diatoms** |
| *Corethron hystrix* Hensen |
| *Coscinodiscus* sp. 1* |
| *Coscinodiscus* sp. 2* |
| *Cyclotella choctawhatcheeana*Prasad |
| *Cylindrotheca closterium* (Ehrenberg) Reimann & J.C.Lewin * |
| *Dactyliosolen fragilissimus* (Bergon) Hasle |
| *Dactyliosolen phuketensis* (B.G.Sundström) G.R.Hasle |
| *Detonula pumila* (Castracane) Gran* |
| *Ditylum brightwellii*(T.West) Grunow* |
| *Entomoneis* sp.* |
| *Eucampia cornuta* (Cleve) Grunow* |
| *Eucampia* sp. |
| *Fragilaria* spp. |
| *Fragilariopsis doliolus* (Wallich) Medlin & P.A.Sims* |
| *Gossliera tropica* Schütt* |
| *Guinardia delicatula* (Cleve) Hasle |
| *Guinardia flaccida* (Castracane) H.Peragallo* |
| *Gyrosigma* sp.* |
| *Haslea* spp.* |
| *Haslea wawrikae* (Hustedt) R.Simonsen |
| *Hemiaulus hauckii* Grunow ex Van Heurck |
| *Lennoxia faveolata* H.A.Thomsen & K.R.Buck |
| *Leptocylindrus danicus* Cleve |
| *Leptocylindrus mediterraneus* (H.Peragallo) Hasle |
| *Lioloma* sp.* |
| *Meuniera membranacea*(Cleve) P.C.Silva |
| *Navicula distans* (W.Smith) Ralfs* |
| *Navicula* spp. |
| *Neocalyptrella robusta* (G.Norman ex Ralfs) Hernández-Becerril & Meave del Castillo |
| *Neodelphineis indica* (F.J.R.Taylor) Y.Tanimura |
| *Nitzschia bicapitata* Cleve |
| *Nitzschia braarudii* Hasle |
| *Nitzschia longissima* (Brébisson) Ralfs |
| *Nitzschia sicula* (Castracane) Hustedt |
| *Nitzschia* spp. |
| *Odontella longicruris* (Greville) M.A.Hoban* |
| *Plagiotropis* spp. |
| *Planktoniella sol* (G.C.Wallich) Schütt |
| *Pleurosigma* sp. |
| *Podosira* sp.* |
| *Proboscia alata* (Brightwell) Sundström |
| *Pseudo-nitzschia delicatissima* (Cleve) Heiden |
| *Pseudo-nitzschia pseudodelicatissima* (Hasle) Hasle |
| *Pseudo-nitzschia seriata* (Cleve) H.Peragallo* |
| *(Continued on next page)* |
| **Diatoms** |
| *Pseudosolenia calcar-avis*(Schultze) B.G.Sundström |
| *Rhizosolenia castracanei* H.Peragallo* |
| *Rhizosolenia clevei* Ostenfeld |
| *Rhizosolenia clevei var. communis* Sundström* |
| *Rhizosolenia fallax* B.G.Sundström* |
| *Rhizosolenia formosa* H.Peragallo* |
| *Rhizosolenia hebetata f. semispina*(Hensen) Gran |
| *Rhizosolenia imbricata* Brightwell |
| *Rhizosolenia setigera f. pungens* (A.Cleve) Brunel* |
| *Skeletonema sp.* |
| *Striatella* sp.* |
| *Thalassionema bacillare* (Heiden) Kolbe |
| *Thalassionema frauenfeldii* (Grunow) Tempère & Peragallo* |
| *Thalassionema nitzschioides* (Grunow) Mereschkowsky |
| *Thalassionema* spp. |
| *Thalassiosira* spp. |
| *Thalassiothrix* spp. |
| *Tropidoneis* sp.* |
| Other unidentified diatoms (<20µm) |
| **Dinoflagellates** |
| *Ceratocorys* sp.* |
| *Chrysocromulina* sp. |
| *Dinophysis acuminata* Claparède & Lachmann* |
| *Dinophysis* sp.* |
| *Diplopsalis* sp.* |
| *Gonyaulax* spp.* |
| *Gymnodinium* spp. |
| *Gyrodinium* spp. |
| *Karenia* sp. |
| *Oxytoxum* spp.* |
| *Oxytoxum variabile* Schiller |
| *Oxytoxum sphaeroideum* Stein |
| *Oxytoxum milneri* Murray & Whitting |
| *Phalacroma rotundatum* (Claparéde & Lachmann) Kofoid & Michener* |
| *Phalacroma* sp.* |
| *Podolampas elegans* Schütt* |
| *Podolampas palmipes* Stein* |
| *Podolampas* sp.* |
| *Prorocentrum balticum* (Lohmann) Loeblich* |
| *Prorocentrum compressum* (J.W.Bailey) Abé ex J.D.Dodge * |
| *Prorocentrum micans* Ehrenberg* |
| *Prorocentrum rostratum* Stein |
| *Protoperidinium bipes* (Paulsen) Balech* |
| *(Continued on next page)* |
|  |
| **Dinoflagellates** |
| *Protoperidinium* spp. |
| *Scrippsiella* sp. |
| *Tripos arietinus* (Cleve) F.Gómez* |
| *Tripos azoricus*(Cleve) F.Gómez* |
| *Tripos carriensis* (Gourret) F.Gómez* |
| *Tripos concilians* (Jørgenen) F.Gómez* |
| *Tripos extensum* (Gourret) F.Gómez* |
| *Tripos furca* (Ehrenberg) F.Gómez |
| *Tripos fusus* (Ehrenberg) F.Gómez |
| *Tripos lineatum* (Ehrenberg) F.Gómez* |
| *Tripos macroceros* (Ehrenberg) F.Gómez* |
| *Tripos massiliensis*(Gourret) F.Gómez |
| *Tripos muelleri* Bory* |
| *Tripos pentagonum* (Gourret) F.Gómez* |
| *Tripos pulchellus*(Schröder) F.Gómez* |
| *Tripos* spp.* |
| *Tripos symmetricus* (Pavillard) F.Gómez* |
| *Tripos teres* (Kofoid) F.Gómez |
| Other unidentified dinoflagellates (<20µm) |
| **Coccolithophores** |
| *Acanthoica quattrospina* Lohmann* (SEM) |
| *Calcidiscus leptoporus subsp. quadriperforatus* (Kamptner) Geisen* (SEM) |
| *Calciosolenia brasiliensis* (Lohmann) J.R.Young (SEM) |
| *Calciosolenia corsellii* Malinverno* (SEM) |
| *Calciosolenia murrayi Gran* (SEM) |
| *Calciosolenia* spp.* |
| *Calyptrosphaera galea* Lecal-Schlauder* (SEM) |
| *Calyptrosphaera oblonga* Lohmann (SEM) |
| *Calyptrosphaera* sp.* |
| *Coronosphaera mediterranea* (Lohmann) Gaarder* (SEM) |
| *Discosphaera tubifera* (Murray & Blackman) Ostenfeld (SEM) |
| *Emiliania huxleyi type A* Young & Westbroek* (SEM) |
| *Emiliania huxleyi type B*Young & Westbroek* (SEM) |
| *Florisphaera profunda*Okada & Honjo* (SEM) |
| *Gephyrocapsa ericsonii* McIntyre & Bé* (SEM) |
| *Gephyrocapsa ericsonii protohuxleyi* type Cros & Fortuño* (SEM) |
| *Gephyrocapsa muellerae* Bréhéret* (SEM) |
| *Helicosphaera carteri* (Wallich) Kamptner* (SEM) |
| *Helicosphaera* spp.* |
| *Michaelsarsia adriatica* (Schiller) Manton, Bremer & Oates (SEM) |
| *Michaelsarsia elegans* Gran* (SEM) |
| *Ophiaster formosus* Gran *** (SEM) |
| *Ophiaster hydroideus*(Lohmann) Lohmann* (SEM) |
| *Ophiaster* sp. |
| *Polycrater sp.** (SEM) |
| *Rhabdosphaera stylifera* Lohmann |
| *Rhabdosphaera xiphos* (Deflandre & Fert) Norris* (SEM) |
| *Scyphosphaera* *apsteinii*Lohmann* |
| *Syracosphaera anthos* (Lohman) Janin* (SEM) |
| *Syracosphaera bannockii* (Borsetti & Cati) Cros* (SEM) |
| *Syracosphaera corolla*J.Lecal* (SEM) |
| *Syracosphaera dilatata* Jordan* (SEM) |
| *Syracosphaera halldalii*HOL Gaarder ex R.W.Jordan & J.C.Green* (SEM) |
| *Syracosphaera hirsuta* Kleijne & Cros* (SEM) |
| *Syracosphaera marginaporata* M.Knappertsbusch* (SEM) |
| *Syracosphaera molischii* type 2 Young* (SEM) |
| *Syracosphaera molischii* Schiller HOL* (SEM) |
| *Syracosphaera nana*(Kamptner) Okada & McIntyre* (SEM) |
| *Syracosphaera nodosa* Kamptner* (SEM) |
| *Syracosphaera ossa* type 2 Young* (SEM) |
| *Syracosphaera pulchra* Lohmann (SEM) |
| *Syracosphaera rotula* Okada & McIntyre* (SEM) |
| *Syracosphaera* sp.* |
| *Umbellosphaera irregularis* Paasche* (SEM) |
| *Umbellosphaera tenuis* (Kamptner) Paasche* (SEM) |
| *Umbilicosphaera foliosa (Kamptner ex Kleijne) Geisen** (SEM) |
| *Umbilicosphaera hulburtiana*Gaardner* (SEM) |
| Other unidentified Coccolithophores (<20µm) |
| ***Cryptophyceae*** |
| ***Cyanobacteria*** |
| *Richelia intracelularis* J.A.Schmidt |
| **Other autotrophs** |
| *Chrysocromulina* sp. |
| *Dictyocha fibula* Ehrenberg |
| *Eutreptia* sp. |
| *Meringosphaera mediterranea* Lohmann |
| *Micromonas* sp. |
| *Phaeocystis* sp. |
| Other unidentified phytoflagellates (<20µm) |
| **Other heterotrophs** |
| *Ebria tripartita* (J.Schumann) Lemmermann* |
| *Globigerina* spp.* |
| *Radiolaria* sp.* |
| *Rhabdonellopsis* sp.* |
| Other unidentified heterotrophs (<20 µm) |
