# Supplementary material for: Phytoplankton diversity and chemotaxonomy in contrasting North Pacific ecosystems
Source: PeerJ. 2023 Jan 3;11:e14501. doi: 10.7717/peerj.14501 (PMC9817951; doi:10.7717/peerj.14501)
Supplement: Supplemental Information 3 — R statistic shows value between 0 (no difference between ranks) and 1 (difference between ranks). Bold values show groups that are different with statistic significance. [file peerj-11-14501-s003.docx]

Table S2. Results of pairwise ANOSIM R test between groups ST1, ST2 and ST3. R statistic shows value between 0 (no difference between ranks) and 1 (difference between ranks). Bold values show groups that are different with statistic significance.

| Groups | R Statistic | Significance Level % | Possible Permutations | Actual Permutations | Number Observed |
| --- | --- | --- | --- | --- | --- |
| ST1, ST2 | 0,218 | 0,7 | 43758 | 999 | 6 |
| **ST1, ST3** | **0,579** | 0,1 | 44352165 | 999 | 0 |
| **ST2, ST3** | **0,612** | 0,1 | 4292145 | 999 | 0 |
