# Supplementary material for: Phytoplankton diversity and chemotaxonomy in contrasting North Pacific ecosystems
Source: PeerJ. 2023 Jan 3;11:e14501. doi: 10.7717/peerj.14501 (PMC9817951; doi:10.7717/peerj.14501)
Supplement: Supplemental Information 4 — Variable names: alloxanthin (Allo), zeaxanthin (Zea), divinyl chlorophyll b (DvChl b), divinyl chlorophyll a (DVChl a), lutein (Lut), diadino, perdinin (Perid), and diatoxanthin (Diato). Sample statistic (Rho): 0,532. Significance level of sample statistic: 1%. Number of permutations: 99. [file peerj-11-14501-s004.docx]

**Table S3.** BEST Global test. Results show Spearman rank correlation factor for each number of permuted variables. Variable names: alloxanthin (Allo), zeaxanthin (Zea), divinyl chlorophyll *b* ( Dv_Chl_*b*), divinyl chlorophyll *a* (DV_Chl_*a*), lutein (Lut), diadino, perdinin (Perid), and diatoxanthin (Diato). Sample statistic (Rho): 0,532. Significance level of sample statistic: 1%. Number of permutations: 99.

| Number Of Variables | Spearman Rank Correlation | Variables |
| --- | --- | --- |
| 4 | 0,532 | [Allo],[Zea],[DV_Chl_b],[Lut] |
| 5 | 0,526 | [Allo], [Diadino], [Zea], [DV_Chl_b], [Lut] |
| 5 | 0,525 | [Allo], [Perid], [Zea], [DV_Chl_b], Abs[Lut] |
| 5 | 0,525 | [Tot_Chl_b], [Allo], [Zea], [DV_Chl_b], [Lut] |
| 4 | 0,524 | [Tot_Chl_b], [Allo], [Zea], [DV_Chl_b] |
| 6 | 0,524 | [Tot_Chl_b], [Allo], [Diadino], [Zea], [DV_Chl_b], [Lut] |
| 4 | 0,522 | [Tot_Chl_b], [Allo], [DV_Chl_b], [Lut] |
