# Supplementary material for: Phytoplankton diversity and chemotaxonomy in contrasting North Pacific ecosystems
Source: PeerJ. 2023 Jan 3;11:e14501. doi: 10.7717/peerj.14501 (PMC9817951; doi:10.7717/peerj.14501)
Supplement: Supplemental Information 5 [file peerj-11-14501-s005.docx]

**Table S4.** Pigment concentration (in µg/L) averaged by stations.

| SAMPLE NAME | | Station 1 | Station 2 | Station 3 |
| --- | --- | --- | --- | --- |
| **PRIMARY** | Total chlorophyll *a* ^1^ | 0.189 | 0.141 | 0.768 |
|  | Total chlorophyll *b* ^2^ | 0.050 | 0.026 | 0.098 |
|  | Total chlorophyll *c* ^3^ | 0.037 | 0.034 | 0.174 |
|  | Carotenes | 0.023 | 0.013 | 0.034 |
|  | 19'-butanoyloxyfucoxanthin | 0.023 | 0.023 | 0.047 |
|  | 19'-hexanoyloxyfucoxanthin | 0.037 | 0.036 | 0.149 |
|  | Alloxanthin |  |  | 0.047 |
|  | Diadinoxanthin | 0.008 | 0.007 | 0.062 |
|  | Diatoxanthin | 0.001 | 0.001 | 0.006 |
|  | Fucoxanthin | 0.006 | 0.005 | 0.122 |
|  | Peridinin | 0.002 | 0.002 | 0.014 |
|  | Zeaxanthin | 0.064 | 0.045 | 0.037 |
| **SECONDARY** | Monovinyl chlorophyll *a* | 0.088 | 0.079 | 0.742 |
|  | Divinyl chlorophyll *a* | 0.100 | 0.061 | 0.012 |
|  | Chlorophyllide *a* | 0.001 | 0.001 | 0.020 |
|  | Monovinyl chlorophyll *b* | 0.019 | 0.012 | 0.097 |
|  | Divinyl chlorophyll *b* | 0.032 | 0.014 | 0.004 |
|  | Chlorophyll *c_1_* + chlorophyll *c_2_* + MGDVP^4^ | 0.017 | 0.015 | 0.099 |
|  | Chlorophyll *c_3_* | 0.020 | 0.019 | 0.075 |
| **TERTIARY** | Lutein | 0.001 |  | 0.003 |
|  | Neoxanthin | 0.001 | 0.001 | 0.011 |
|  | Violaxanthin | 0.001 | 0.001 | 0.023 |
|  | Total pheophytin *a* | 0.002 | 0.001 | 0.007 |
|  | Total pheophorbide *a* |  | 0.001 | 0.017 |
|  | Prasinoxanthin | 0.001 | 0.001 | 0.032 |
